# The Optimization of the Transition Zone of the Planar Heterogeneous Interface for High-Performance Seawater Desalination

**DOI:** 10.3390/ma15103561

**Published:** 2022-05-16

**Authors:** Chang Liu, Hui Liu, Pengfei Ma, Yan Liu, Ruochong Cai, Ran Yin, Biao Zhang, Shiqi Wei, Huifang Miao, Liuxuan Cao

**Affiliations:** 1 College of Energy, Xiamen University, Xiamen 361005, China; 32420191152345@stu.xmu.edu.cn (C.L.); 32420201152831@stu.xmu.edu.cn (H.L.); 32420191152349@stu.xmu.edu.cn (P.M.); 32420201152803@stu.xmu.edu.cn (Y.L.); 32420182202900@stu.xmu.edu.cn (R.C.); 32420192202854@stu.xmu.edu.cn (R.Y.); 32420192202856@stu.xmu.edu.cn (B.Z.); weishiqi@stu.xmu.edu.cn (S.W.); 2 Fujian Provincial Nuclear Energy Engineering Technology Research Center, Xiamen 361005, China

**Keywords:** desalination, low energy consumption, two-dimensional material, heterojunction

## Abstract

Reverse osmosis has become the most prevalent approach to seawater desalination. It is still limited by the permeability-selectivity trade-off of the membranes and the energy consumption in the operation process. Recently, an efficient ionic sieving with high performance was realized by utilizing the bi-unipolar transport behaviour and strong ion depletion of heterogeneous structures in 2D materials. A perfect salt rejection rate of 97.0% and a near-maximum water flux of 1529 L m^−2^ h^−1^ bar^−1^ were obtained. However, the energy consumption of the heterogeneous desalination setup is a very important factor, and it remains largely unexplored. Here, the geometric-dimension-dependent ion transport in planar heterogeneous structures is reported. The two competitive ion migration behaviours during the desalination process, ion-depletion-dominated and electric-field-dominated ion transport, are identified for the first time. More importantly, these two ion-transport behaviours can be regulated. The excellent performance of combined high rejection rate, high water flux and low energy consumption can be obtained under the synergy of voltage, pressure and geometric dimension. With the appropriate optimization, the energy consumption can be reduced by 2 orders of magnitude, which is 50% of the industrial energy consumption. These findings provide beneficial insight for the application and optimized design of low-energy-consumption and portable water desalination devices.

## 1. Introduction

The shortage of freshwater is a severe challenge for humans and is predicted to worsen in the future owing to growing industrialization, rapid population growth and urbanization [1,2,3]. To address the lack of freshwater resources, the utilization of unconventional water sources, such as seawater and wastewater, to increase water supply is urgently demanded as the ultimate solution [4,5]. For the desalination process, the energy consumption is an important factor because most of the cost is attributable to energy consumption [6,7]. Membrane-based technologies have gradually gained considerable attention because they are more environmentally friendly and show lower specific energy consumption compared with the other competitive technologies [8,9,10,11]. In particular, reverse osmosis is currently the most prevalent option because the specific energy consumption has been significantly reduced from about 10 kWh/m^3^ to 3~4 kWh/m^3^ [12,13,14,15]. Under the thermodynamic limit of 0.9 kWh/m^3^, there is still great potential for further development [8]. However, reverse osmosis is still limited by the permeability-selectivity trade-off, which means the high throughput and perfect rejection rate cannot be achieved together [16,17].

Emerging two-dimensional materials provide a promising solution to the permeability-selectivity trade-off in reverse osmosis membranes [18,19,20,21]. Two-dimensional membranes (2DMs) present atomic thickness with low flow resistance and energy loss for precise ionic and molecular sieving in aqueous solution, with applications in desalination and wastewater treatment [22,23,24]. In addition, the energy and space barrier induced by the two-dimensional structure and its tuneable physicochemical properties [25,26,27] can effectively block most of the ions in the solution and generate a high rejection rate [28,29]. Several strategies are proposed for excluding ions of hydrated diameter no more than 9 Å [30], including cation control [31], capillary compression [32], physical confinement [9] and partial reduction [33]. However, the sub-nanometre dimension in 2DMs greatly hinders water flux lower than 0.1 L m^−2^ h^−1^ bar^−1^, which markedly limits industrial application [9,34].

In addition to size-based repulsion, more novel transport mechanisms at nano- or sub-nano scale could be utilized to realize ion sieving or separation, including ion concentration polarization [35,36], asymmetric ion transport [37,38,39] and heterogeneous membrane structure [40,41,42,43,44]. These phenomena can exist at scales larger than 1 nm, which effectively enhance the permeability while maintaining the ion rejection. In this regard, these novel transport mechanisms are considered to be hopeful approaches to resolving the permeability-selectivity trade-off. Recently, Wei Guo’s group reported efficient ionic sieving in a planar heterogeneous graphene oxide membrane with coupled electric fields and hydraulic pressure [45]. Utilizing the bi-unipolar transport behaviour of heterogeneous structures and the strong ion depletion state in the near-neutral transition zone under certain applied electric fields, the conduction of both cations and anions is blocked. In this way, the device obtains a perfect salt rejection rate of 97.0% and a near-maximum water flux of 1529 L m^−2^ h^−1^ bar^−1^ without adjusting the interlayer distance to sub-nanometre scale. The process provides a new route to realizing high-performance desalination. In-depth and systematic research on the optimization of seawater desalination processes, such as the ion rejection rate, water flux and energy consumption, is very important but remains largely unexplored.

Herein, we systematically investigate the role of the transition zone in planar heterogeneous interface desalination with a special focus on the energy consumption. Based on the combined Navier-Stokes (NS) equations and Poisson-Nernst-Planck (PNP) equations, we study the influence factor of geometric dimension, the electric field and hydraulic pressure on the ion rejection rate, the water flux and the energy consumption. The results indicate that although the enlarged interlayer distance enhances the water flux, it also undermines the ionic rejection rate. In particular, the size of the transition zone can significantly determine the performance of seawater desalination, including the ion rejection rate and the water flux. Theoretical analysis reveals two competitive factors governing the ionic transport. With the enlargement of the transition region, the ion transport in the planar heterogeneous interface shifts from ion-depletion-dominated ion sieving to electric-field-governed desalination. Intriguingly, the unit energy consumption can achieve the minimum with the optimization of the transition zone size. Through adjusting the geometric dimension of the transition zone, combined with other optimized parameters, high salt rejection rates of more than 90% and high water flux can be achieved. The system power consumption decreases by about 90% and is only 25~50% of the existing industrial pure water energy consumption level [46,47], representing a feasible advance toward practical applications.

## 2. Materials and Methods

### 2.1. Numerical Calculation

Ionic transport in planar heterogeneous 2D nanochannels driven by the electric field and hydraulic pressure was analysed under the framework of continuum dynamics. The coupled Navier-Stokes (NS) equations and Poisson-Nernst-Planck (PNP) equations were used to calculate the ion transport behaviour. The heterojunction consisted of three parts, the positively charged part (P-part), the negatively charged part (N-part) and the charge-neutral transition zone in the middle (Figure S1). A 2D model was employed to simulate the interlayer space of the 2D lamellas. The interlayer distance *D* was set to be 2 nm, which ensured the accuracy of the continuous approximation and was close to the interlayer distance of the 2D membranes [48]. The surface charge densities of the N-part and P-part were set to −0.06 C/m^−2^ and +0.06 C/m^−2^, respectively [49]. The solution on both sides of the model was 100 mM NaCl to simulate real seawater [50,51]. The coupled differential equations with appropriate boundary conditions were solved through the finite element method using COMSOL commercial calculation software. The ion concentration, electric potential and water flux in the systems were obtained.

The ion transport in 2D nanochannels is analysed under the framework of continuum dynamics. The combined Poisson-Nernst-Planck (PNP) equations and the Navier-Stokes (NS) equations are used to calculate the ionic transport in nanoscale [52,53]. The PNP equation is as follows:

(1) $$\vec{j_{i}}=D_{i}\left(\nabla c_{i}+\frac{z_{i}ec_{i}}{k_{B}T}\nabla \Phi \right)$$

(2) $$\nabla^{2}\Phi =-\frac{e}{\varepsilon }\left(c_{+}-c_{-}\right)$$

where *i* stands for the ionic species, $\vec{j_{i}}$ is the local ionic flux, *c_i_* is the local ion concentration, *D_i_* is the diffusion coefficient, *z_i_* is the valence, and Φ is the local electrical potential. The variables *k_B_*, *e*, *ε* and *T* represent the Boltzmann constant, the dielectric constant of the electrolyte solution, the electron charge and the temperature, respectively.

The NS equation is as follows [54]:

(3) $$\vec{u}\cdot \nabla \vec{u\;}=\frac{1}{\rho }\left[-\nabla p+\mu \nabla^{2}\vec{u}-\left(c_{+}-c_{-}\right)\nabla \Phi \right]=0\;\;\;\;\;i=+/-$$

where $\vec{u}$ stands for the fluid velocity, ρ is the density of solution, *p* is the pressure and μ is the viscosity of solution.

The ionic flux satisfies the continuity equation,

(4) $$\nabla \cdot \vec{j_{i}}=0$$

The boundary condition for Φ is governed by Gauss’s law [55],

(5) $$\vec{n}\cdot \nabla \Phi =-\frac{\sigma_{s}}{\varepsilon_{0}\varepsilon_{r}}$$

where *ε_0_* and *ε_r_* denote the permittivity of the vacuum and the relative dielectric constant of the electrolyte solution, respectively. $\varepsilon_{r}$ = *80*. $\vec{n}$ stands for the unit vector in the normal direction. The spacing charge density (*σ_s_*) can be described as [45],

(6) $$\sigma_{s}=F\left(z_{+}c_{+}+z_{-}c_{-}\right)$$

where F is the Faraday constant, 96,485 C/mol.; *z_+_* and *z_−_* are the charge numbers of cations and anions, respectively; and *c_+_* and *c_−_* are the concentrations of cations and anions, respectively.

The local ionic flux (*j_i_*) should have zero normal components at boundaries:

(7) $$\vec{n}\cdot \vec{j_{i}}=0$$

The finite element method was used to solve the coupled partial differential equations with appropriate boundary conditions. In this way, the electric potential distribution, the local ion concentration and the resulting ionic flux can be obtained. The total ionic current contributed separately by cations (*I_+_*) or anions (*I_−_*) can be calculated by integrating the ionic flux in the cross-section of the model channel [48],

(8) $$I_{i}=2\pi e\int Dj_{i}dr\;\;\;\;\;i=+/-$$

where *D* is the radial size of the 2D channel.

### 2.2. Models Parameters

Details of the calculation model can be found in Table 1. Grid independence analysis was carried out to achieve the necessary calculation accuracy (Figure S2).

**Table 1:** The parameters of the calculation models.

Parameter	Description	Value	Parameters Involved in the Manuscript
*D*	Interlayer distance	2 nm	Figure 1 and Figure 2, Figures 4 and 5,
			Figures S1–S3, Figures S6–S9
		2, 4, 6, 8 10 nm	Figure 3
		2, 6, 10 nm	Figure S4
		2, 10 nm	Figure S5
*L*	Length of nanochannel	100 mM	Figure 1, Figure 2, Figure 3, Figure 4 and Figure 5, Figures S1–S9
*C*_0_	Electrolyte concentration	100 mM	Figure 1, Figure 2, Figure 3, Figure 4 and Figure 5, Figures S1–S9
*σ*	Surface charge density	±0.06 C/m^2^	Figure 1, Figure 2, Figure 3, Figure 4 and Figure 5, Figures S1–S9
*e_L_*	Length of one-side charge region	49 nm	Figure 1, Figure 2 and Figure 3, Figures S1–S5
		49, 30, 20, 10, 2 nm	Figure 4, Figure 5, Figure S7
		47.5, 10, 2 nm	Figure 5
		30 nm, 2 nm	Figures S8 and S9
		49, 40, 30, 20, 10, 2 nm	Figure S6
*d*	Length of the transition zone	2 nm	Figure 1, Figure 2 and Figure 3, Figures S1–S5
		2, 40, 60, 80, 96 nm	Figure 4 and Figure 5, Figure S7
		5, 80, 96 nm	Figure 5
		40 nm, 96 nm	Figures S8 and S9
		2, 20, 40, 60, 80, 96 nm	Figure S6
*D_p_*	Diffusion coefficient of Na^+^	1.344 × 10^−5^ cm^2^ s^−1^	Figure 1, Figure 2, Figure 3, Figure 4 and Figure 5, Figures S1–S9
*D_n_*	Diffusion coefficient of Cl^−^	2.032 × 10^−5^ cm^2^ s^−1^	Figure 1, Figure 2, Figure 3, Figure 4 and Figure 5, Figures S1–S9
Δ*U*	Voltage	0.2 V	Figure 1, Figure 3, Figure 5, Figure S1–S9
		0.1, 0.2, 0.3, 0.4, 0.5 V	Figure 2,
		0.1, 0.2, 0.5 V	Figure 4,
Δ*P*	Pressure	0.1 MPa	Figure 1, Figure 3, Figure 4 and Figure 5, Figures S1–S9
		0.02, 0.04, 0.06, 0.08, 0.1 MPa	Figure S2

**Figure 1:**
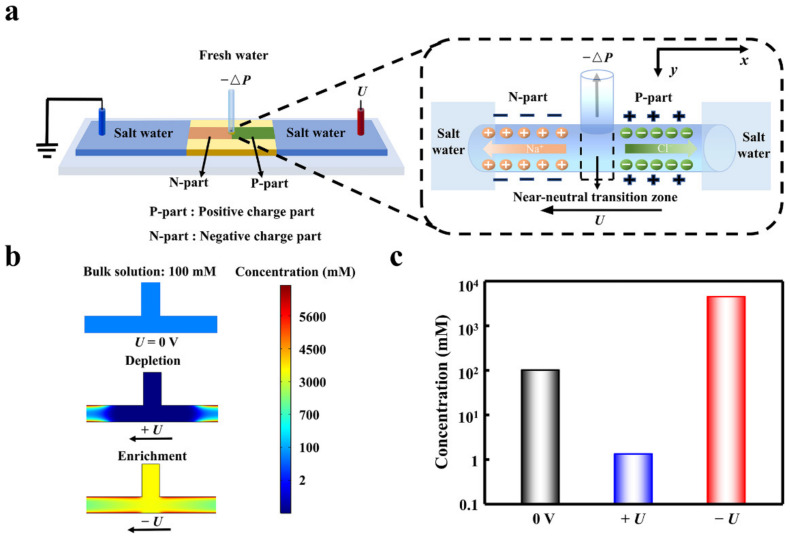
The desalination process in the planar heterogeneous interface. (**a**) Scheme of the heterogeneous desalination setup. (**b**) The voltage-dependent ion depletion and enrichment in the transition zone. The ion depletion effect under forward bias makes the ion concentration obviously lower than that of the enrichment state and the bulk solution. (**c**) The evident desalination can be achieved under ion depletion state assisted by the forward voltage. The voltages were set to be 0.2 V and −0.2 V.

**Figure 2:**
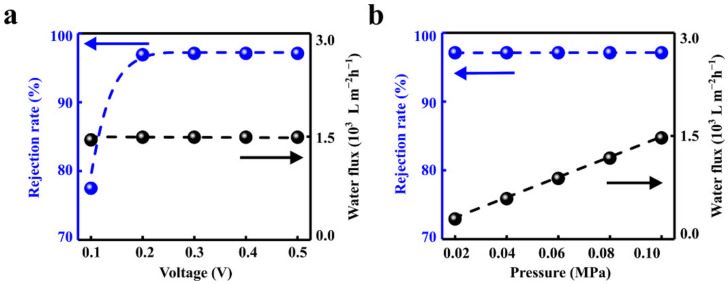
Effects of electric field and hydraulic pressure. (**a**) The increased voltage can significantly enhance the rejection rate while the water flux remains stable. (**b**) The water flux can be facilitated linearly with the hydraulic pressure while maintaining the high ion rejection rate.

**Figure 3:**
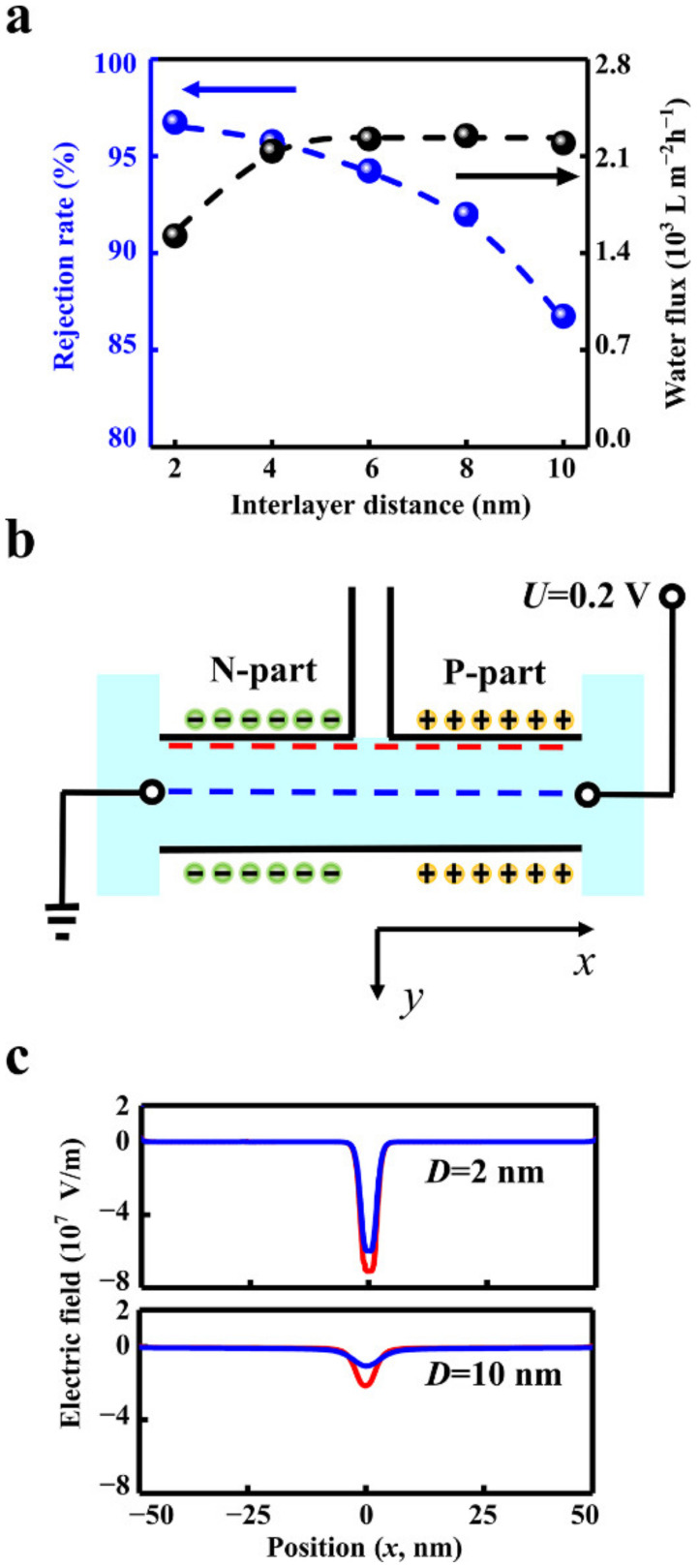
The influence of interlayer distance. (**a**) The increased interlayer distance (*D*) degrades the salt rejection rate (blue line) and enhances the water flux (black line). (**b**) The electric fields along the blue and red lines were extracted to indicate the electric barrier. (**c**) The absolute value of the electric field intensity with the interlayer distance of 2 nm is about 3 times higher than that of 10 nm.

**Figure 4:**
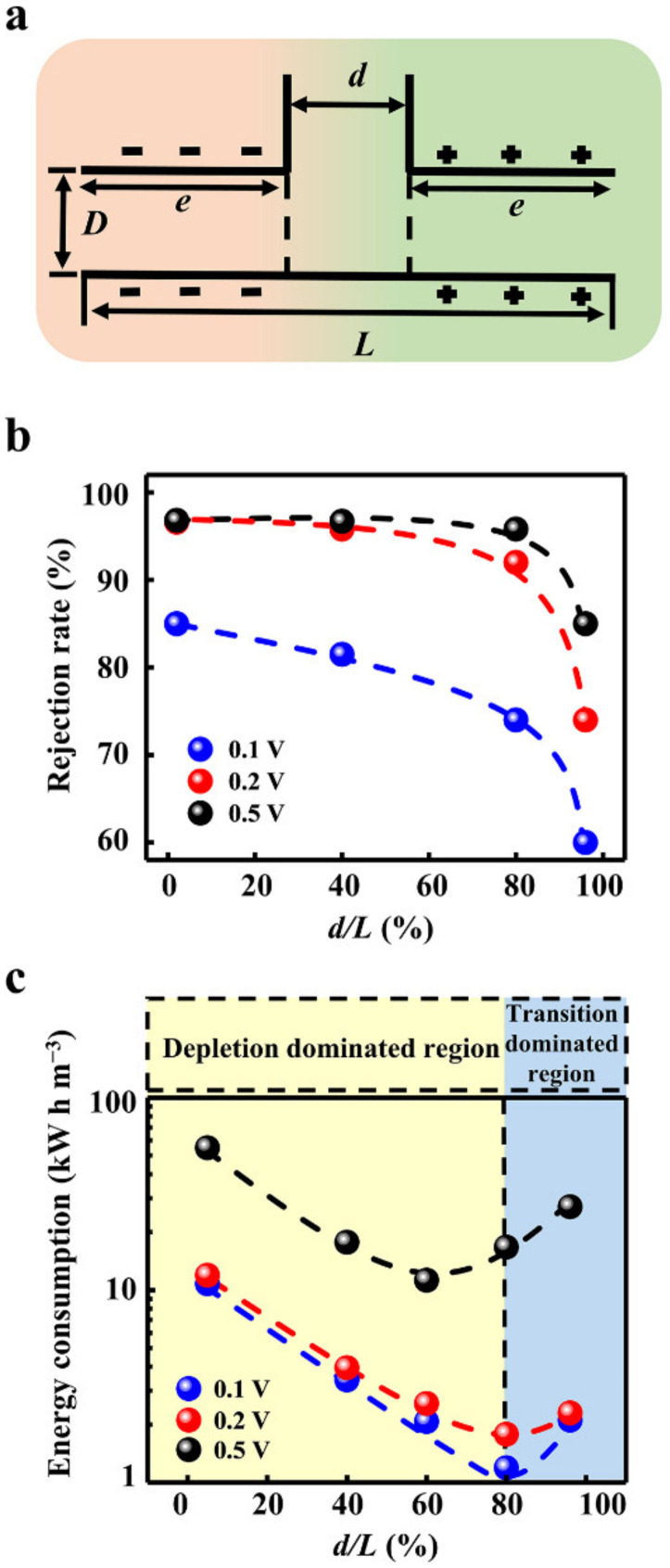
Influence of the geometric dimensions of the transition zone. (**a**) Schematic diagram of the geometric parameters. (**b**) The increment of the transition zone reduces the rejection rate. (**c**) The ion rejection generated by the applied voltage instead of the intrinsic charge separation and potential barrier leads to high electric energy consumption. Through the optimization of the geometric dimensions, the lowest energy consumption can be obtained.

**Figure 5:**
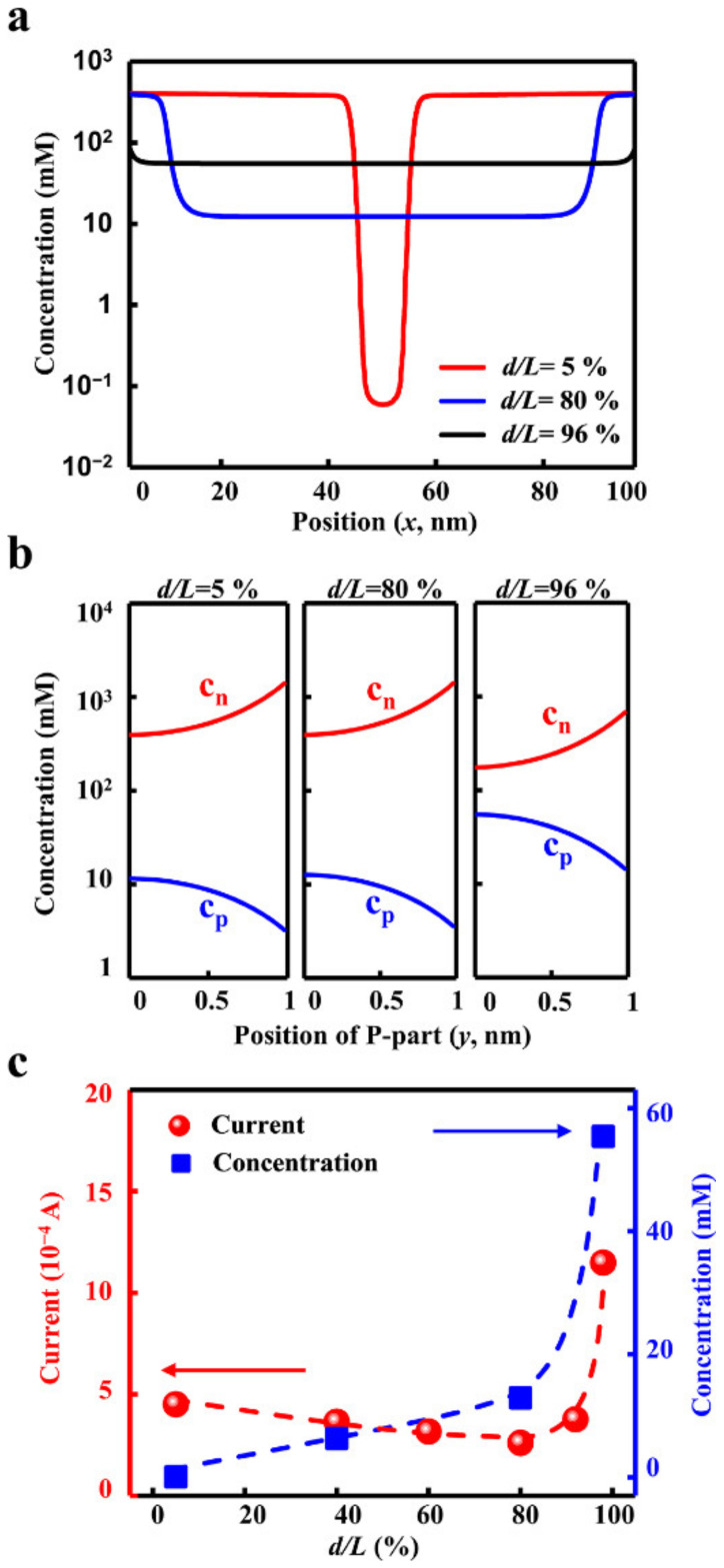
The mechanism. (**a**) The axial distribution of the ion concentrations under different transition zone sizes. (**b**) The radial distributions of cations (c_p_) and anions (c_n_) indicate the charge separation effect. (**c**) The ion concentration rises with the size of the transition zone. The ionic current decreases with the transition zone and reaches the minimum. When the transition zone widens larger than 80%, the current enhances sharply.

## 3. Results and Discussion

Assisted by a forward electric field, ion depletion occurs in the transition zone, and the deionized water can be extracted from the depletion zone (Figure 1a). The total ion concentration in the transition area is an order of magnitude lower than the bulk concentration (Figure S3a). For comparison, the ion concentration is higher than the bulk under the reverse bias. The transition zone plays the important role in the ion depletion effect. The cations are attracted in the left negatively charged part owing to the electrostatic interaction, while the anions are attracted in the right positively charged part (Figure S3b,c). The forward bias forms a high potential barrier of 2 × 10^6^ V m^−1^ in the transition zone, which prevents both the anions and the cations from passing through the transition zone. This results in a low-conducting state in the transition zone. In sharp contrast, the ion can easily enter the transition zone under the reverse bias. Therefore, the depletion effect under forward bias makes the ion concentration obviously lower than that of the enrichment state and the bulk solution (Figure 1b). Compared with the bulk concentration, the ion concentration in the transition zone can be suppressed (*+U*) and enhanced (*−U*) 100 times, respectively (Figure 1c).

The rejection rate and water flux can be regulated by the applied electric field and the hydraulic pressure. The rejection rate is positively correlated with the voltage and eventually reaches saturation of 96.89% when the applied voltage is larger than 0.2 V (Figure 2a). This is in accord with the literature reports [45,56,57]. The water flux increases linearly with hydraulic pressure, which is consistent with the results in the literature [58]. When the hydraulic pressure increases from 0 to 0.1 MPa, the outlet water flux increases linearly from 0 to 1492 L m^−2^ h^−1^. The salt rejection rate in the solution remains stable (Figure 2b).

The interlayer distance of the planar structure can affect the ion rejection rate and the water flux. The calculation results show that when the interlayer distance increases from 2 nm to 10 nm, the rejection rate reduces notably from 96.9% to 86.8% (Figure 3a). The water flux at the outlet enhances from 1492 L m^−2^ h^−1^ to 2195 L m^−2^ h^−1^ when the interlayer distance increases from 2 nm to 6 nm, then reaching a plateau. The plateau is produced by the limitation of fluid velocity in the outlet (Figure S4a.). When the interlayer distance increases to more than 6 nm, the fluid velocity in the outlet remains stable (Figure S4b). Limited by the total water flux in the outlet, the fluid velocity in the inlet decreases with the increment of the interlayer distance (Figure S4c). Thus, the interlayer distance should match the size of the outlet to obtain high water flux.

A too-large interlayer distance is unable to enhance the water flux and degrades the rejection rate. The potential barrier in the transition zone dominates the ion rejection effect. The increased interlayer distance weakens the potential barrier. The electric field distribution profile along the central line zone demonstrates the details (Figure 3b). The absolute value of the electric field intensity in the transition zone with the interlayer distance of 2 nm is about three times higher than that of 10 nm (Figure 3c). The higher electric field strength in transition zone leads to the lower ion concentration (Figure S5) and eventually promotes the ion rejection effect.

The outlet size is directly related to the water flux. In the calculation model, the width of the outlet is set to be the same as the size of the near-neutral transition zone (Figure 4a). The inlet and outlet fluxes are equal, obeying the law of conservation of mass (Figure S6). As expected, the larger outlet significantly increases the water flux (Figure S7). Intriguingly, the outlet size can markedly influence the rejection rate. We fix the total length of the calculation model and change the proportion of the transition zone from 2% to 96%. When the transition zone increases, the decline in rejection rate occurs in all applied voltage conditions (Figure 4b). In particular, when the applied voltage is relatively low (0.2 V), the rejection rate decreases below 90% with a transition zone larger than 80%. Though the higher voltage can enhance the ion rejection, the rejection rate also drops sharply if the transition zone increases further.

The degradation of the rejection rate stems from the weakened ion depletion in the large transition zone. The ion concentration of the short transition zone (Figure S8a) is significantly lower than that of the long transition zone (Figure S8b). The potential barrier is weakened by the enlarged transition zone (Figure S8c). Meanwhile, the charge separation is also degraded in the long transition zone (Figure S8d). These factors contributed to the boost in the ion concentration in the outlet (Figure S9).

Though the enhanced voltage can apparently promote the ion rejection rate, even when the transition zone occupies a large proportion, the ion rejection generated by the applied voltage instead of the intrinsic charge separation and potential barrier will lead to high electric energy consumption (Figure 4c). For instance, the energy consumption to obtain per unit volume of pure water under 0.5 V is about 10 times higher than that at 0.1 V or 0.2 V. Furthermore, the energy consumption strongly depends on the width of the transition zone. As the size of the transition zone increases, the energy consumption gradually decreases and then reaches the minimum value. Consequently, there is an optimized geometry design for obtaining the lowest energy consumption. For the low applied voltage, the larger transition zone can be adopted to gain both the high water flux and low energy consumption. To minimize the power consumption while balancing the high rejection rate, the transition zone can be designed to occupy 80%, and the working voltage can be set as 0.2 V. In this case, the lowest energy consumption of 1.78 kW h/m^3^ and the rejection rate of above 90% can be realized together, which is only 50% of the energy consumption of the current seawater desalination industry [46,47].

The optimized transition zone for achieving the low consumption originates from the transition-zone-dependent water flux and ion depletion effect. On the one hand, the enlarged transition zone allows the low flow resistance, which leads to the low energy consumption. On the other hand, the oversized transition zone undermines the ion depletion and the charge separation. Thus, with the expansion of the transition zone, the ion transport in the planar heterogeneous channels is gradually dominated by the electric field instead of the ion depletion effect.

Importantly, the different ionic transport behaviours mainly rule the energy efficiency of the desalination process. The enlarged transition zone degrades the ion depletion, which leads to the obvious boost in the ion concentration. As shown in Figure 5a, when *d/L* increases from 5% to 80%, the ion concentration grows from about 0.1 mM to 10 mM, with 2 orders of magnitude. When the transition zone expands further, the ion concentration increases to about 100 mM. The high ion concentration weakens the charge separation along the radial direction (Figure 5b), which accordingly undermines the ion selectivity. Owing to the high ionic conductance induced by the degradation of the ion depletion, the current across the planar heterojunction enhanced sharply (Figure 5c). These two competitive factors, the ion-depletion-dominated and electric-field-dominated ion transport, result in the minimum energy consumption in the optimized geometry design.

## 4. Conclusions

In conclusion, we investigate the role of the transition zone in the planar heterogeneous interface in seawater desalination with special focus on the optimization of energy consumption. The results show that the geometric dimensions of the heterojunction region are critical for the desalination process. The excellent performance of high rejection rate, high water flux and low energy consumption combined can be obtained under the synergism of voltage, pressure and transition zone size. A larger transition zone reduces rejection and increases water flux, stemming from the distinct ion transport property in the planar heterogeneous interface. The two essential desalination processes governed by the geometric dimensions, ion depletion and the electric-field-dominated ion migration property, are identified for the first time. These can be utilized to increase the ion rejection rate and reduce the energy consumption. With the appropriate optimization, the energy consumption can be reduced by two orders of magnitude while maintaining the ion rejection rate above 90%, which is 50% of the industrial level. These findings provide beneficial insights for the application and optimized design of low-energy-consumption and portable water desalination devices.

## Data Availability

The data presented in this study are available in Supplementary Materials here.

## Supplementary Materials

The following supporting information can be downloaded at: https://www.mdpi.com/article/10.3390/ma15103561/s1, Figure S1: Schematic of the calculation model, Figure S2: Grid sensitivity analysis, Figure S3: The mechanism of desalination, Figure S4: The outlet size limits the increment of water flow caused by the expansion in interlayer distance, Figure S5: The ion concentration in the larger interlayer is higher, Figure S6: The inlet and outlet fluxes obey the law of conservation of mass, Figure S7: Calculation model of water flux, Figure S8: The length of the transition zone affects ion separation and EDL, Figure S9: Three-dimensional ion concentration distribution.

## Nomenclature

2DMs	two-dimensional membranes
EDL	electric double layer
NS	Navier–Stokes
PNP	Poisson–Nernst–Planck
*c_i_*	local ion concentration
*c_+_*	concentration of cations
*c_-_*	concentration of anions
*D_i_*	diffusion coefficient
*e*	dielectric constant of the electrolyte solution
F	Faraday constant
*i*	ionic species
$j_{i}$	local ionic flux
*k_B_*	Boltzmann constant
$\vec{n}$	the unit vector in normal direction
*p*	pressure
*T*	temperature
$\vec{u}$	fluid velocity
*z_i_*	valence
*z_+_*	charge number of cations
*z_-_*	charge number of anions
*ε*	electron charge
*ε_0_*	permittivity of vacuum
*ε_r_*	relative dielectric constant of the electrolyte solution
μ	viscosity of solution
ρ	density of solution
Φ	local electrical potential

## References

[1] Grant S.B., Saphores J.-D., Feldman D.L., Hamilton A.J., Fletcher T.D., Cook P.L., Stewardson M., Sanders B.F., Levin L.A., Ambrose R.F.J.S. (2012). Taking the “waste” out of “wastewater” for human water security and ecosystem sustainability. Science.

[2] Tang J.Y.M., Busetti F., Charrois J.W.A., Escher B.I. (2014). Which chemicals drive biological effects in wastewater and recycled water?. Water Res..

[3] Liu H., Huang Z., Liu K., Hu X., Zhou J. (2019). Interfacial Solar-to-Heat Conversion for Desalination. Adv. Energy Mater..

[4] Werber J.R., Osuji C.O., Elimelech M. (2016). Materials for next-generation desalination and water purification membranes. Nat. Rev. Mater..

[5] Dudchenko A.V., Chen C., Cardenas A., Rolf J., Jassby D. (2017). Frequency-dependent stability of CNT Joule heaters in ionizable media and desalination processes. Nat. Nanotechnol..

[6] Lin S. (2020). Energy Efficiency of Desalination: Fundamental Insights from Intuitive Interpretation. Environ. Sci. Technol..

[7] Hepburn C., Qi Y., Stern N., Ward B., Xie C., Zenghelis D. (2021). Towards carbon neutrality and China’s 14th Five-Year Plan: Clean energy transition, sustainable urban development, and investment priorities. Environ. Sci. Ecotechnol..

[8] Elimelech M., Phillip W.A. (2011). The Future of Seawater Desalination: Energy, Technology, and the Environment. Science.

[9] Abraham J., Vasu K.S., Williams C.D., Gopinadhan K., Su Y., Cherian C.T., Dix J., Prestat E., Haigh S.J., Grigorieva I.V. (2017). Tunable sieving of ions using graphene oxide membranes. Nat. Nanotechnol..

[10] Amy G., Ghaffour N., Li Z., Francis L., Linares R.V., Missimer T., Lattemann S. (2017). Membrane-based seawater desalination: Present and future prospects. Desalination.

[11] Zhang Z., Shen W., Lin L., Wang M., Li N., Zheng Z., Liu F., Cao L. (2020). Vertically Transported Graphene Oxide for High-Performance Osmotic Energy Conversion. Adv. Sci..

[12] Ghaffour N., Missimer T.M., Amy G.L. (2013). Technical review and evaluation of the economics of water desalination: Current and future challenges for better water supply sustainability. Desalination.

[13] Alkaisi A., Mossad R., Sharifian-Barforoush A. (2017). A review of the water desalination systems integrated with renewable energy. Energy Procedia.

[14] Lim Y.J., Goh K., Kurihara M., Wang R. (2021). Seawater desalination by reverse osmosis: Current development and future challenges in membrane fabrication—A review. J. Membr. Sci..

[15] Ihsanullah I., Atieh M.A., Sajid M., Nazal M.K. (2021). Desalination and environment: A critical analysis of impacts, mitigation strategies, and greener desalination technologies. Sci. Total Environ..

[16] Voutchkov N. (2018). Energy use for membrane seawater desalination–current status and trends. Desalination.

[17] Lin S., Zhao H., Zhu L., He T., Chen S., Gao C., Zhang L. (2021). Seawater desalination technology and engineering in China: A review. Desalination.

[18] Pendergast M.M., Hoek E.M.J.E. (2011). A review of water treatment membrane nanotechnologies. Science.

[19] Ma P., Zheng J., Zhao D., Zhang W., Lu G., Lin L., Zhao Z., Huang Z., Cao L. (2021). The Selective Transport of Ions in Charged Nanopore with Combined Multi-Physics Fields. Materials.

[20] Imbault A., Wang Y., Kruse P., Strelcov E., Comini E., Sberveglieri G., Kolmakov A. (2015). Ultrathin gas permeable oxide membranes for chemical sensing: Nanoporous Ta_2_O_5_ test study. Materials.

[21] Kim I.I., Kihm K.D. (2015). Nano sensing and energy conversion using surface plasmon resonance (SPR). Materials.

[22] Morelos-Gomez A., Cruz-Silva R., Muramatsu H., Ortiz-Medina J., Araki T., Fukuyo T., Tejima S., Takeuchi K., Hayashi T., Terrones M. (2017). Effective NaCl and dye rejection of hybrid graphene oxide/graphene layered membranes. Nat. Nanotechnol..

[23] Xiao F., Ji D., Li H., Tang J., Feng Y., Ding L., Cao L., Li N., Jiang L., Guo W. (2018). Simulation of osmotic energy conversion in nanoporous materials: A concise single-pore model. Inorg. Chem. Front..

[24] Qu R., Zeng X., Lin L., Zhang G., Liu F., Wang C., Ma S., Liu C., Miao H., Cao L. (2020). Vertically-Oriented Ti_3_C_2_T_x_ MXene Membranes for High Performance of Electrokinetic Energy Conversion. ACS Nano.

[25] Cao L., Wang Y. (2009). Fabrication and investigation of single track-etched nanopore and its applications. Radiat. Measur..

[26] Qiu L., Zhang X., Yang W., Wang Y., Simon G.P., Li D. (2011). Controllable corrugation of chemically converted graphene sheets in water and potential application for nanofiltration. Chem. Commun..

[27] Tseng S., Li Y.M., Lin C.Y., Hsu J.P. (2016). Salinity gradient power: Influences of temperature and nanopore size. Nanoscale.

[28] Xie X., Crespo G.A., Mistlberger G., Bakker E. (2014). Photocurrent generation based on a light-driven proton pump in an artificial liquid membrane. Nat. Chem..

[29] Zhang X., Wen Q., Wang L., Ding L., Yang J., Ji D., Zhang Y., Jiang L., Guo W. (2019). Asymmetric Electrokinetic Proton Transport through 2D Nanofluidic Heterojunctions. ACS Nano.

[30] Jia P., Wen Q., Liu D., Zhou M., Jin X., Ding L., Dong H., Lu D., Jiang L., Guo W. (2019). Highly Efficient Ionic Photocurrent Generation through WS2-Based 2D Nanofluidic Channels. Small.

[31] Chen L., Shi G., Shen J., Peng B., Zhang B., Wang Y., Bian F., Wang J., Li D., Qian Z. (2017). Ion sieving in graphene oxide membranes via cationic control of interlayer spacing. Nature.

[32] Yang X., Cheng C., Wang Y., Qiu L., Li D. (2013). Liquid-Mediated Dense Integration of Graphene Materials for Compact Capacitive Energy Storage. Science.

[33] Su Y., Kravets V.G., Wong S.L., Waters J., Geim A.K., Nair R.R. (2014). Impermeable barrier films and protective coatings based on reduced graphene oxide. Nat. Commun..

[34] Park H.B., Kamcev J., Robeson L.M., Elimelech M., Freeman B.D. (2017). Maximizing the right stuff: The trade-off between membrane permeability and selectivity. Science.

[35] Kim S.J., Ko S.H., Kang K.H., Han J. (2010). Direct seawater desalination by ion concentration polarization. Nat. Nanotechnol..

[36] Cao L., Xiao F., Feng Y., Zhu W., Geng W., Yang J., Zhang X., Li N., Guo W., Jiang L. (2017). Anomalous Channel-Length Dependence in Nanofluidic Osmotic Energy Conversion. Adv. Funct. Mater..

[37] Guo W., Tian Y., Jiang L. (2013). Asymmetric Ion Transport through Ion-Channel-Mimetic Solid-State Nanopores. Acc. Chem. Res..

[38] Li H., Xiao F., Hong G., Su J., Li N., Cao L., Wen Q., Guo W. (2019). On the Role of Heterogeneous Nanopore Junction in Osmotic Power Generation. Chin. J. Chem..

[39] Zhang Z., Wang C., Lin L., Xu M., Wu Y., Cao L. (2020). Rectified Ion Transport in Ultra-thin Membrane Governed by Outer Membrane Electric Double Layer. Chin. J. Chem..

[40] Han Y., Xu Z., Gao C. (2013). Ultrathin Graphene Nanofiltration Membrane for Water Purification. Adv. Funct. Mater..

[41] Feng Y., Zhu W., Guo W., Jiang L. (2017). Bioinspired Energy Conversion in Nanofluidics: A Paradigm of Material Evolution. Adv. Mater..

[42] Ji D., Wen Q., Cao L., Kang Q., Lin S., Zhang X., Jiang L., Guo W. (2019). Electrokinetically Controlled Asymmetric Ion Transport through 1D/2D Nanofluidic Heterojunctions. Adv. Mater. Technol..

[43] Wang L., Wen Q., Jia P., Jia M., Lu D., Sun X., Jiang L., Guo W. (2019). Light-Driven Active Proton Transport through Photoacid- and Photobase-Doped Janus Graphene Oxide Membranes. Adv. Mater..

[44] Zhang Y., Li F., Kong X., Xue T., Liu D., Jia P., Wang L., Ding L., Dong H., Lu D. (2020). Photoinduced Directional Proton Transport through Printed Asymmetric Graphene Oxide Superstructures: A New Driving Mechanism under Full-Area Light Illumination. Adv. Funct. Mater..

[45] Wen Q., Jia P., Cao L., Li J., Quan D., Wang L., Zhang Y., Lu D., Jiang L., Guo W. (2020). Electric-Field-Induced Ionic Sieving at Planar Graphene Oxide Heterojunctions for Miniaturized Water Desalination. Adv. Mater..

[46] Al-Karaghouli A., Kazmerski L.L. (2013). Energy consumption and water production cost of conventional and renewable-energy-powered desalination processes. Renew. Sustain. Energ. Rev..

[47] Zarzo D., Prats D. (2018). Desalination and energy consumption. What can we expect in the near future?. Desalination.

[48] Cao L., Wen Q., Feng Y., Ji D., Li H., Li N., Jiang L., Guo W. (2018). On the Origin of Ion Selectivity in Ultrathin Nanopores: Insights for Membrane-Scale Osmotic Energy Conversion. Adv. Funct. Mater..

[49] Ji J., Kang Q., Zhou Y., Feng Y., Chen X., Yuan J., Guo W., Wei Y., Jiang L. (2017). Osmotic Power Generation with Positively and Negatively Charged 2D Nanofluidic Membrane Pairs. Adv. Funct. Mater..

[50] Adham S., Hussain A., Matar J.M., Dores R., Janson A. (2013). Application of Membrane Distillation for desalting brines from thermal desalination plants. Desalination.

[51] Millero F.J., Feistel R., Wright D.G., McDougall T.J. (2008). The composition of Standard Seawater and the definition of the Reference-Composition Salinity Scale. Deep Sea Res..

[52] Ai Y., Qian S. (2011). Electrokinetic particle translocation through a nanopore. Phys. Chem. Chem. Phys..

[53] Gao J., Guo W., Feng D., Wang H., Zhao D., Jiang L. (2014). High-performance ionic diode membrane for salinity gradient power generation. J. Am. Chem. Soc..

[54] Wen Q., Yan D., Liu F., Wang M., Ling Y., Wang P., Kluth P., Schauries D., Trautmann C., Apel P.J.A.F.M. (2016). Highly selective ionic transport through subnanometer pores in polymer films. Adv. Funct. Mater..

[55] Astar W. (1998). Applications of multidimensional distributions in electrostatics. IEEE Trans. Educ..

[56] Zhen-Peng G.E., Yan-Chao S.H.I., Xiao-Yi L.I. (2013). Effects of Orthogonal Electric Field on Water Flux through a Carbon Nanotube. Acta Phys.-Chim. Sin..

[57] Shahryari M., Nazari-Golshan A., Nourazar S.S. (2022). The study of heat flux and external electric field effects on carbon nanotube behavior as an atomic nano-pump. App. Phys. A.

[58] Thomas M., Corry B. (2016). A computational assessment of the permeability and salt rejection of carbon nanotube membranes and their application to water desalination. Philos. Trans. A Math. Phys. Eng. Sci..

