# Supplementary material for: The Optimization of the Transition Zone of the Planar Heterogeneous Interface for High-Performance Seawater Desalination
Source: Materials (Basel). 2022 May 16;15(10):3561. doi: 10.3390/ma15103561 (PMC9143191; doi:10.3390/ma15103561)
Supplement: Supplementary file 1 [file materials-15-03561-s001.zip › materials-1706006-supplementary.pdf]

Supporting Information

The Optimization towards Transition Zone of Planar Heterogeneous Interface for High-performance Seawater Desalination

Chang Liu[1], Hui Liu[1], Pengfei Ma[1], Yan Liu[1], Ruochong Cai[1], Ran Yin[1], Biao Zhang[1], Shiqi Wei[1], Huifang Miao[1,2,*], Liuxuan Cao[1,2,*]

[1] College of Energy, Xiamen University, Xiamen, Fujian 361005, P. R. China.

[2] Fujian Provincial Nuclear Energy Engineering Technology Research Center, Xiamen, Fujian 361005, P.R. China.

*Corresponding authors.

E-mail: caoliuxuan@xmu.edu.cn (L. Cao)

E-mail: hfmiao@xmu.edu.cn (H. Miao)

Table of the content:

1. Calculation model.

2. Grid sensitivity analysis.

3. Mechanism of desalination.

4. Influence of interlayer distance.

5. Influence of the transition zone size.

6. References.

1. Calculation Model

The calculation model is shown in Figure S1. 2D model was employed to simulate the transport of ions in the planar 2D nanochannels. Two solution reservoirs are connected by a charged channel (L=100 nm, D=2 nm). The solution reservoirs are of the same size, 2 μm×4 μm. The charge densities σ of the N-part and P-part are −0.06 C/m^2 and +0.06 C/m^2, respectively. [1] The length of one-side charged region is e_L. In the middle is the near-neutral transition zone with no charge. The length of transition zone is d. The electrolyte concentration in solution reservoirs is C_0. The diffusion coefficient of Na^+ and Cl^- are D_p and D_n, respectively. The interlayer distance D =2 nm. A negative pressure $\triangle P$ is applied on the outlet. And the applied external voltage is $\triangle U$.

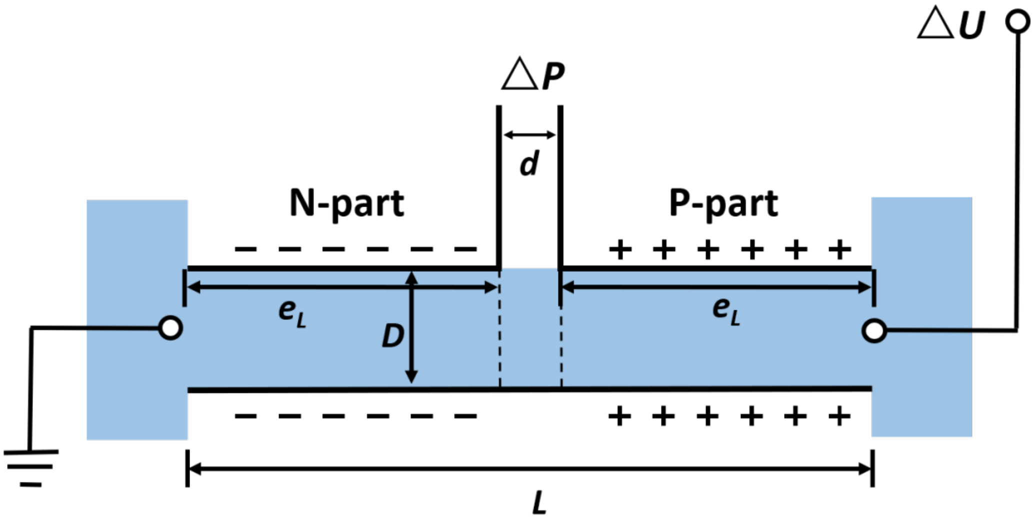

Figure S1. Schematic of the calculation model. The 2D axisymmetric model contains P-part, N-part, and a transition zone in between.

2. Grid Sensitivity Analysis

Smaller and denser grids make the calculation results more accurate, but induce more computing resources and time. To balance the calculation accuracy and calculation cost, the grid sensitivity analysis was carried out. Three equidistant positions on the central axis were selected to analyze the corresponding concentration and velocity under different grid numbers. As shown in Figure S2, when the number of grids is below 60,000, the calculation results change obviously with the number of grids. When the number of grids exceeds 90,000, the calculation results converge to one platform. In this case, the change of the grid number influences the calculation results no longer. Therefore, the grid number of our calculation were all set to larger than 90,000.

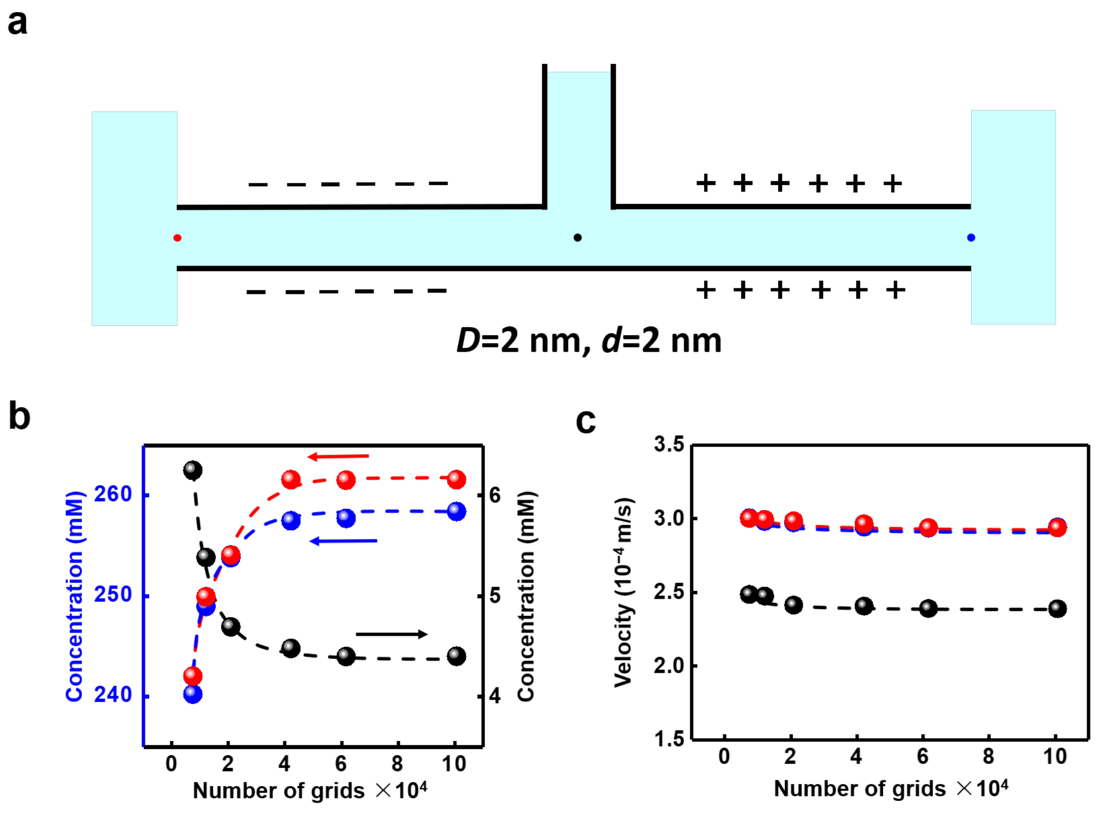

Figure S2. Grid sensitivity analysis. (**a**) Three testing points with equal distance on the central axis were selected to test the calculation concentration and velocity under different grid numbers. (**b**) The calculated ion concentration changes with the number of grids. (**c**) The velocity corresponding to the three points varies with the number of grids.

3. Mechanism of Desalination

The ion depletion and enrichment effects of heterojunction channels is essential for the ion desalination process. Assisted by the forward bias, the total ionic concentration in the transition area is almost an order of magnitude lower than the bulk concentration, indicating the strong ion depletion state. In this case, the salt rejection rate can be higher than 90 %. For comparison, the ion concentration is higher than the bulk value under the reverse bias (Figure S3a). The ion can easily enter the transition zone under the reverse bias, forming an enrichment effect (Figure S3b). The forward bias forms a high potential barrier in the transition zone, which prevents both the anions and cations from passing through the transition zone. It results in a low-conducting state in the transition zone (Figure S3c).

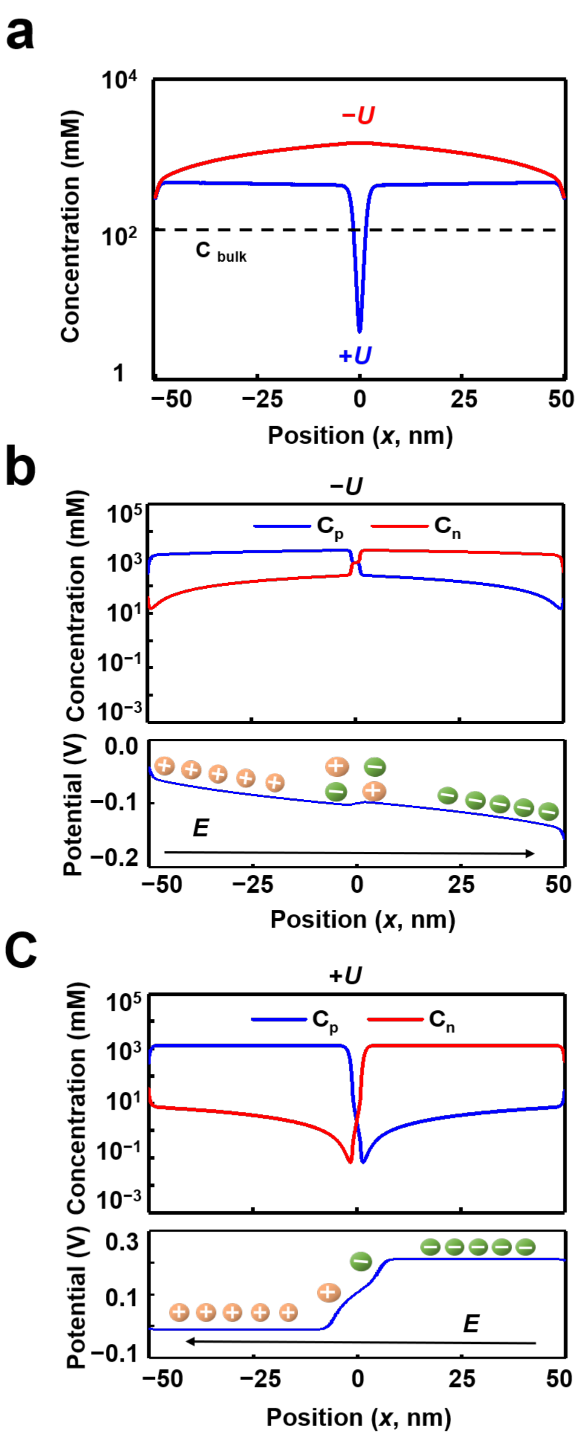

Figure S3. The mechanism of desalination. (**a**) The total ion concentration distribution along the central axis reveals the desalination stems from the voltage-polarity-dependent ion depletion in the transition zone. (**b**) Ionic concentration profile (top) and electric potential distribution (bottom) under the enrichment state. (**c**) The mechanism of salt-ion blockade in the transition zone under the depleted state. The bulk concentration was 100 mM.

4. Influence of Interlayer Distance

The water flows in the heterojunction from both sides and is drawn out from the outlet in the middle (Figure S4a). Due to the limitation of the outlet size, when the interlayer distance increases to larger than 6 nm, the outlet flow velocity reaches the maximum (Figure S4b). If the outlet size keeps invariant, the flow in the interlayer will keep stable. As the interlayer distance increases further, the water flow velocity in the interlayer will decrease (Figure S4c).

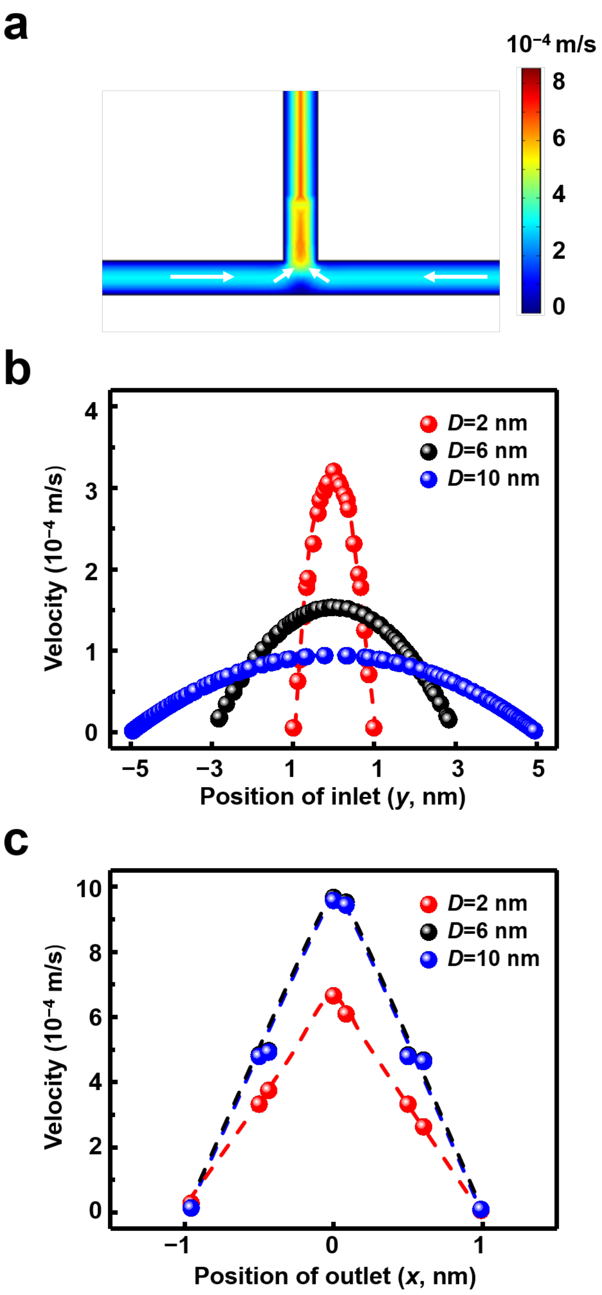

Figure S4. The outlet size limits the increment of water flow caused by the expansion in interlayer distance. (**a**) The water flow distribution. (**b**) Limited by the outlet size, the outlet

flow velocity will not increase after the interlayer distance enlarges to a certain size. (**c**) As the size of outlet increases, the flows velocity of inlet decreases.

Too larger interlayer distance is unable to enhance the water flux, but degrade the rejection rate. The increased interlayer distance weakens the potential barrier. The higher electric field strength in smaller transition zone leads to the lower ion concentration and eventually raises the ion rejection effect. The ion concentration in the transition zone with the interlayer distance of 10 nm is about 3~4 times higher than that of 2 nm (Figure S5).

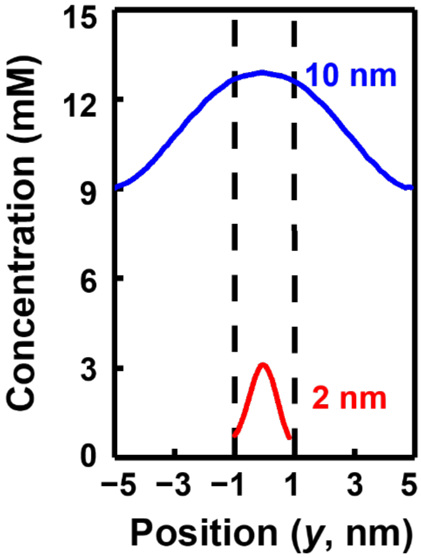

Figure S5. The ion concentration in larger interlayer is higher.

5. Influence of the Transition Zone Size

The fluxes of inlet and outlet are equal, obeying the law of conservation of mass. The increase of the transition zone size makes the inlet flow velocity faster, but the outlet flow velocity change slowly. The ratio of the water flux at the entrance to the outlet is maintained at around 1 (Figure S6).

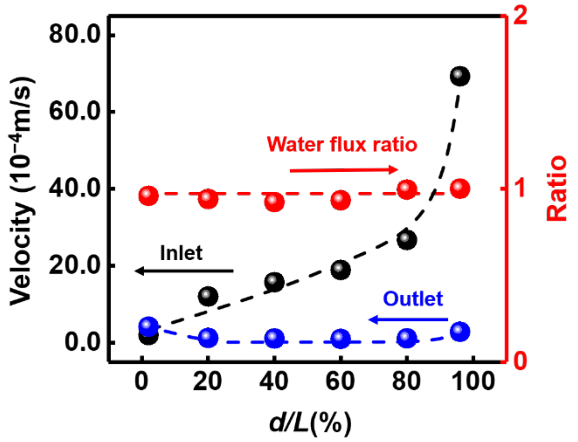

Figure S6. The fluxes of inlet and outlet obeys the law of conservation of mass.

The water flux grows nonlinearly with the expansion of the transition zone. We extend the 2D model in the y-axis direction, and the size of outlet is $d \times h$. The h is the length of the outlet, set to 100 nm (Figure S7a). With the expansion of the transition zone, the water flux also increases (Figure S7b).

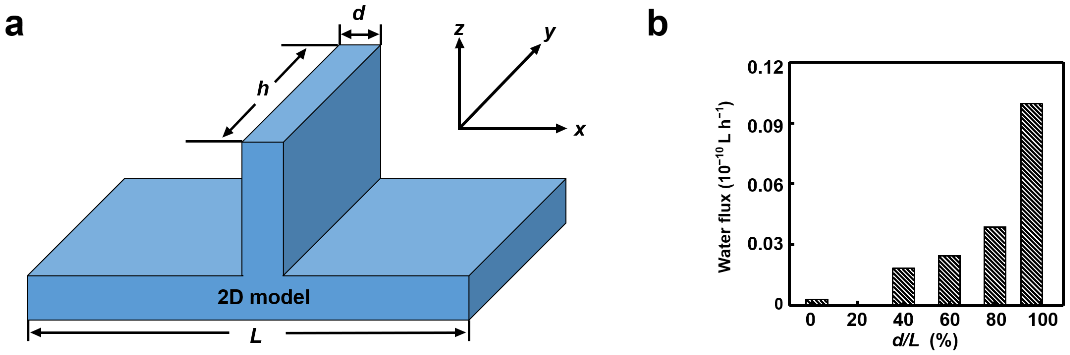

Figure S7. Calculation model of water flux. (**a**) Extending the 2D model to calculate the water flux at the outlet. (**b**)The enlarged transition zone increases the water flux.

The expansion of the transition zone weakens the electrical potential and the effect of EDL. When *d*=40 nm, the anions and cations are blocked in the P-part and the N-part respectively (Figure S8a). When *d*=96 nm, more ions enter the transition zone and enhance the ion strength (Figure S8b). The enlarged transition zone weakens the potential barrier and promotes the ion enrichment in the transition zone (Figure S8c). The increment of the transition zone reduces the area of the EDL, weakening the ion separation (Figure S8d). These factors weaken the ion rejection effect together.

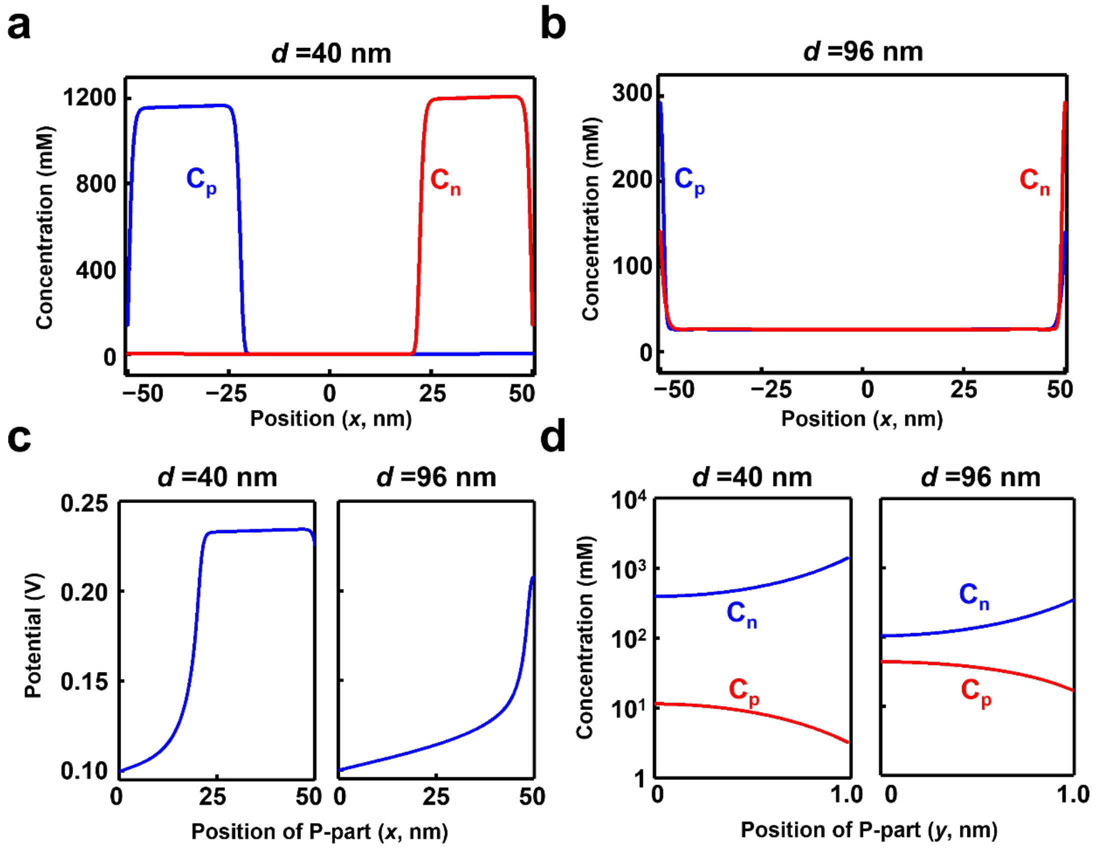

Figure S8. The length of the transition zone affects ion separation and EDL. The axial concentration of anion and cation when *d*=40 nm (**a**) and *d*=96 nm (**b**). (**c**) The narrower transition zone leads to the steeper electric potential distribution. (**d**) The narrower transition zone generates stronger ion separation.

The ion concentration in the transition zone is extremely lower than that of charged zone, with the magnitude of more than 10^3 times (Figure S9a and Figure S9b). When the transition zone increases to 96 nm (Figure S9c), the ion strength enhances obviously. The difference in ion concentration between the transition zone and charged zone is about 50 times, which is much lower than that of smaller transition area (Figure S9d).

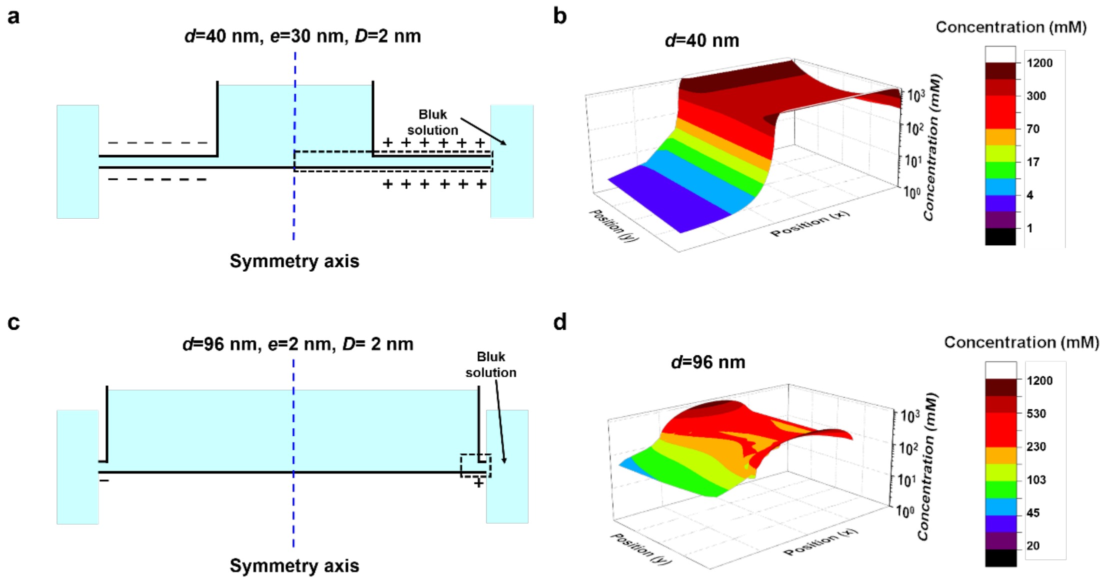

Figure S9. Three-dimensional ion concentration distribution. (**a**) The transition zone is 40 nm. (**b**) The anion concentration in the transition zone is much lower than that of the charged zone. (**c**) The transition zone is 96 nm. (**d**) The anion concentration in the transition zone largely enhances.

6. References

1. Ji, J.; Kang, Q.; Zhou, Y.; Feng, Y.; Chen, X.; Yuan, J.; Guo, W.; Wei, Y.; Jiang, L., Osmotic Power Generation with Positively and Negatively Charged 2D Nanofluidic Membrane Pairs. *Adv. Funct. Mater.* **2017,** 27, 1603623.
